# Application of Machine-Learning Algorithms for Better Understanding the Properties of Liquisolid Systems Prepared with Three Mesoporous Silica Based Carriers

**DOI:** 10.3390/pharmaceutics15030741

**Published:** 2023-02-23

**Authors:** Teodora Glišić, Jelena Djuriš, Ivana Vasiljević, Jelena Parojčić, Ivana Aleksić

**Affiliations:** Department of Pharmaceutical Technology and Cosmetology, Faculty of Pharmacy, University of Belgrade, Vojvode Stepe 450, 11221 Belgrade, Serbia

**Keywords:** liquisolid systems, mesoporous silica carrier, flowability, direct compression, compaction behavior, machine-learning

## Abstract

The processing of liquisolid systems (LSS), which are considered a promising approach to improving the oral bioavailability of poorly soluble drugs, has proven challenging due to the relatively high amount of liquid phase incorporated within them. The objective of this study was to apply machine-learning tools to better understand the effects of formulation factors and/or tableting process parameters on the flowability and compaction properties of LSS with silica-based mesoporous excipients as carriers. In addition, the results of the flowability testing and dynamic compaction analysis of liquisolid admixtures were used to build data sets and develop predictive multivariate models. In the regression analysis, six different algorithms were used to model the relationship between tensile strength (TS), the target variable, and eight other input variables. The AdaBoost algorithm provided the best-fit model for predicting TS (coefficient of determination = 0.94), with ejection stress (ES), compaction pressure, and carrier type being the parameters that influenced its performance the most. The same algorithm was best for classification (precision = 0.90), depending on the type of carrier used, with detachment stress, ES, and TS as variables affecting the performance of the model. Furthermore, the formulations with Neusilin^®^ US2 were able to maintain good flowability and satisfactory values of TS despite having a higher liquid load compared to the other two carriers.

## 1. Introduction

The low solubility of active pharmaceutical ingredients (APIs) in the gastrointestinal tract represents one of the biggest obstacles to their application as solid dosage forms and to achieving the desired therapeutic effects. The number of poorly soluble APIs with pronounced hydrophobic properties is constantly increasing, which emphasizes the importance of developing methods that can improve both the solubility and the release of these APIs from the dosage forms and consequently their bioavailability [1]. Over the years, numerous methods and procedures have been developed to overcome these problems. They are based on different principles such as reducing particle size (micronization, nanonization), changing the solid state of the API (amorphization, preparation of solid dispersions), or improving solubility in gastrointestinal fluids (addition of surfactants and complexing agents, development of lipid-based formulations) [2].

The technology of liquisolid systems (LSS) represents a simple and cost-effective approach to overcome the low bioavailability of poorly soluble drugs [3]. The basic principle of LSS formulation implies that a liquid API or a dispersion of a solid API in a suitable liquid vehicle is converted into a dry, non-adhesive powder using suitable excipients with high absorption capacities (carrier and coating material). The obtained powder admixtures, which are characterized by good flowability and good compaction properties, can be further processed into the desired dosage form—granules, capsules, or tablets [4]. Solid, poorly soluble drugs are the most common candidates for formulation using liquisolid (LS) technology. The presence of these APIs within the LSS in the form of a solution/suspension enables faster and more complete release from the applied dosage form, mainly due to the increase in surface area, improved wettability, and, to some extent, an improvement in solubility [5]. The formulation of LSS is based on a mathematical approach developed by Spireas [6]. The two main formulation factors are the carrier to coating material ratio (R)—the ratio between the amount of carrier (Q) and coating (q) material, and the liquid load factor (L_f_)—the ratio between the amount of liquid (W) and carrier (Q).

The potential of the LS technique to improve the bioavailability of poorly soluble drugs has been extensively researched over the years. In a multitude of in vitro studies, including a wide variety of model drugs, significantly improved dissolution profiles were achieved with LS tablets compared to directly compressed tablets of the same composition or similar commercially available formulations [7,8,9,10,11,12]. The results of in vivo studies indicate an improvement in the rate and extent of absorption, higher plasma concentrations, improved bioavailability, and significantly more pronounced therapeutic effects [13,14,15,16,17,18,19]. However, despite its advantages, the LS technology has yet to find its place in practice. This is mainly due to the limitations related to the amount of liquid phase incorporated into the system and the resulting compaction problems. Namely, a higher amount of the liquid phase, often necessary to disperse the required amount of API, can lead to poor mechanical properties of the obtained LS compacts. In addition to low tensile strength (TS) values, visible defects such as capping, lamination, and chipping often occur, especially when detaching and ejecting the LS compacts from the tableting equipment. The mechanical characteristics of LS tablets, such as friability and resistance to crushing, have been investigated in several studies over the years [5,20,21,22,23]. However, few recent studies have discussed the influence of formulation factors on the behavior of LSS during compaction [24,25,26,27] and a more detailed insight into the complex relationship between the formulation of LSS and the process parameters during all stages of compaction is still lacking. Therefore, the application of this technology requires not only a good understanding of the LS formulation and an accurate calculation of the required amounts of excipients but also an understanding of the tableting process and all its stages.

Machine learning (ML) algorithms have been widely used in various areas of health sciences in the last two decades. Their ability to process large amounts of multidimensional data and interpret the mutual influence of a large number of variables means that they can be used for more efficient analysis of medical data and early diagnosis [28,29,30]. The application of ML in drug delivery has become increasingly prominent with the development of more complex techniques such as nanosystems or various approaches to personalized medicine, including 3D printing [31,32]. Over the years, these tools have found their place in various stages of drug development, from molecule discovery and pre-formulation studies to optimization of final dosage forms and pharmaceutical processes [33,34,35]. ML has been widely used in the development and optimization of solid dosage forms with the idea of developing appropriate predictive models. One of the first studies in this field was conducted by Bourquin [36] and coworkers, who used neural networks to model TS and friability with compression force, dwell time in the compression phase, and the amount of excipients used as input parameters. Similar studies using artificial neural networks aimed to investigate the influence of the type and amount of fillers and lubricants with different APIs on tablet characteristics, including tablet hardness [37,38]. In the research carried out by Khalid et al. [39], different ML methods were used to model the relationship between TS as a dependent variable and some of the process parameters (compression pressure (CP), type of tableting device) and formulation factors (proportion of excipients) as independent variables. In addition, a variety of other process parameters and material properties have been used as input variables in different studies, including diameter, mass, and thickness of compacts, porosity, compression speed, type of polymer used, its concentration, etc. [40,41]. Recent studies have also reported the use of convolutional neural networks for product characterization based on imaging studies [42,43]. To the best of our knowledge, in the field of LSS, only Barmpalexis et al. [44] have used ML tools, more specifically artificial neural networks and genetic programming, to investigate the effects of formulation variables on the dissolution of LSS using aprepitant as a model drug. The results of their study suggested that, compared to classical multilinear regression analysis, ML algorithms performed better at establishing complex relationships between the input variables and the responses studied.

The three excipients that were chosen as carriers in this research are all silica based. Silicates are suitable excipients for the incorporation of large amounts of liquids due to their porous structure. Neusilin^®^ US2 is a synthetic, amorphous magnesium aluminometasilicate with a specific surface area (SSA) of up to 300 m^2^/g. It is a multifunctional excipient that can be used for both direct compression and wet granulation. Eleven different grades of this excipient are commercially available, and they differ in specific density, water content, particle size, and pH of the aqueous dispersion. Neusilin^®^ US2 is one of the most commonly used grades in LSS formulations due to its high SSA value and high absorption capacity. The manufacturer also states that the excipient has excellent compressibility and that its use allows the production of tablets with satisfactory hardness even at low compression loads [45]. Syloid^®^ XDP 3050 and Syloid^®^ XDP 3150 are novel excipients of the mesoporous amorphous silica type specifically developed for the formulation of LSS. Their high porosity, density, and liquid absorption capacity make them suitable for converting the dispersion of the API into a powder with good flowability. The particle size, morphology, and pore structure of these excipients were designed to be suitable for incorporating liquid lipophilic APIs without the need to use solvents or surfactants, as is required for most other carriers. According to the manufacturer, Syloid^®^ carriers also have a significantly higher capsule filling capacity than other previously used carriers, such as colloidal silica. In addition, these carriers are said to allow faster and more complete release of the APIs from solid dosage forms compared to other commercially available carriers [46]. A better understanding of LSS, especially their compression properties, would bring us one step closer to their practical application. Industrial production of LSS would be a highly beneficial approach, using well-known excipients and already established production processes to obtain safe dosage forms that are already well accepted by patients, such as tablets, while improving the bioavailability of poorly soluble drugs, which is one of the main problems the pharmaceutical industry is facing today. The use of predictive models in drug development and production is consistent with some of the principles on which the modern pharmaceutical industry is based. It would be beneficial to save time and resources while maintaining a product of consistent quality.

The aim of this research was to investigate the influence of the formulation factors (R, L_f_, the type of carrier used) and the tableting process on the flowability and behavior of LSS during compaction. Investigated LS admixtures were prepared with three different types of carriers: Syloid^®^ XDP 3050, Syloid^®^ XDP 3150, and Neusilin^®^ US2. ML was used for an in-depth analysis of the obtained data and to model the relationships between different input variables and TS as a mechanical characteristic of LS compacts.

## 2. Materials and Methods

### 2.1. Materials

LSS were prepared using two types of mesoporous silica (Syloid^®^ XDP 3050 and Syloid^®^ XDP 3150, Grace GmbH, Worms, Germany) and synthetic, amorphous magnesium aluminometasilicate (Neusilin^®^ US2, Fuji Chemical Industry Co. Ltd., Toyama, Japan) as carriers. Colloidal silicon dioxide (Aerosil^®^ 200, Evonik Industries AG, Rheinfelden, Germany) was selected as the coating material, and polyethylene glycol 400 (PEG 400, Sigma-Aldrich, Taufkirchen, Germany) was used as the liquid phase.

### 2.2. Preparation of LS Admixtures

The admixtures were prepared according to the procedure described by S. Spireas [6] using a mortar and pestle. The carrier was weighed and transferred to a mortar, and the liquid phase was added in drops. In order to distribute the liquid phase evenly, mixing is done at a speed of one rotation per second for one minute and then left to rest for five minutes so that the absorption of the liquid phase into the carrier particles is complete. The previously weighed coating material was then added, and the admixture was gently stirred for 30 s. Table 1 shows the ratio of excipients used. A total of 38 admixtures were prepared using 3 different types of carriers, by varying the proportion of the liquid phase and carrier to the coating material ratio.

### 2.3. LS Admixtures’ Flowability

The flowability of the prepared LS admixtures was tested using an Erweka flowmeter type GDT (Erweka GmbH, Heusenstamm, Germany). The time for which 20 g of the sample passes through the funnel of the device was measured, and the results were expressed as mass flow rate (g/s). Results are shown as the mean value of three measurements. Bulk and tapped densities were measured using a 100 mL graduated cylinder and a shaking volumeter, STAV 2003 (J. Engelsmann AG, Ludwigshafen, Germany). Carr index (CI) and Hausner ratio (HR) were calculated afterwards according to Equations (1) and (2) [47], respectively, where ρ_t_ is tapped density and ρ_b_ is bulk density:

Carr index (%) = 100 × (ρ_t_ − ρ_b_)/ρ_t_ (1)

HR = ρ_t_/ρ_b_ (2)

### 2.4. Dynamic Compaction Analysis

Dynamic compaction analysis [48] was performed on 25 selected LS admixtures (designated in Table 1) using a laboratory compaction simulator (GTP D series, Gamlen Tableting Ltd., Beckenham, UK), at a compaction speed of 60 mm/min, using 6 mm flat faced punches. Each compact weighed 75 mg and the applied compression load ranged from 250 to 500 kg, with an increment of 50 kg. Admixtures with Syloid^®^ carriers were first exposed to a pre-compression load that was half the amount of the compression load used afterwards. At least three compacts were prepared for each compression load, and results were presented as the mean value. Force-displacement curves generated during different phases of the compaction process were used for further calculations in order to analyze the behavior of LSS during compaction. In order to examine the correlation between the values of these parameters and the TS of LS compacts, the following parameters were monitored: CP, detachment stress (DS), ejection stress (ES), net work of compression (NWC), and elastic recovery (ER).

TS was calculated according to Equation (3) [49]:

σ = 2F/πDt(3)

where σ is TS, F is the force required to break the compact, D is the compact diameter, and t is the out-of-die compact thickness after 24 h.

The force required to break the compact and the compact diameter were measured using the Ewreka TBH 125H tablet hardness tester (Ewreka GmbH, Langen, Germany). The thickness of the compacts was measured with a vernier caliper (Digi plus line, Vogel). TS was calculated for at least three samples, and the obtained results were expressed as the mean value.

CP and DS were calculated using Equations (4) and (5), respectively, where F_c_ is the maximum force recorded during tablet compaction, F_d_ is the maximum force recorded during tablet detachment, and r is the compact radius.

CP = F_c_/r^2^π (4)

DS = F_d_/r^2^π (5)

ES is a parameter that indicates the adhesive properties of the material and was calculated according to Equation (6):

ES = F_e_/πDt (6)

where F_e_ is the maximum force recorded during compact ejection, D is the compact diameter, and t is the compact thickness in the ejection phase.

NWC was calculated as a difference between the total work of compression and elastic work. The total work of compression was calculated from the area under the compression force-displacement curve, while the elastic work was calculated from the area under the curve in the decompression phase, and the trapezoidal method was used in both cases. ER was calculated using tablet thickness at maximum compression force (h_max_) and 24 h after compression (h_min_), according to Equation (7). The thickness of the compact 24 h after compression was measured with a vernier caliper (Digi plus line, Vogel).

ER (%) = (h_max_ − h_min_)/h_max_ × 100% (7)

### 2.5. Data Analysis

The analysis of the obtained data was performed using Orange software version 3.31.1 (Bioinformatics Laboratory, Faculty of Computer and Information Science, University of Ljubljana, Ljubljana, Slovenia) [50]. The selection of methods used in this study was based on a review of the available literature and the most commonly used algorithms in the field of pharmaceutical technology. The influence of formulation factors (R, L_f_ and the type of carrier used) on the flowability of the LS admixtures was examined first. The analyzed data set consisted of 6 variables with 39 entries each (Table 1). Pearson’s correlation was used to test the presence of correlations between variables, while different types of graphs and a decision tree were used for data visualization. Data correlation was also assessed through principal component analysis (PCA).

**Figure 1:**
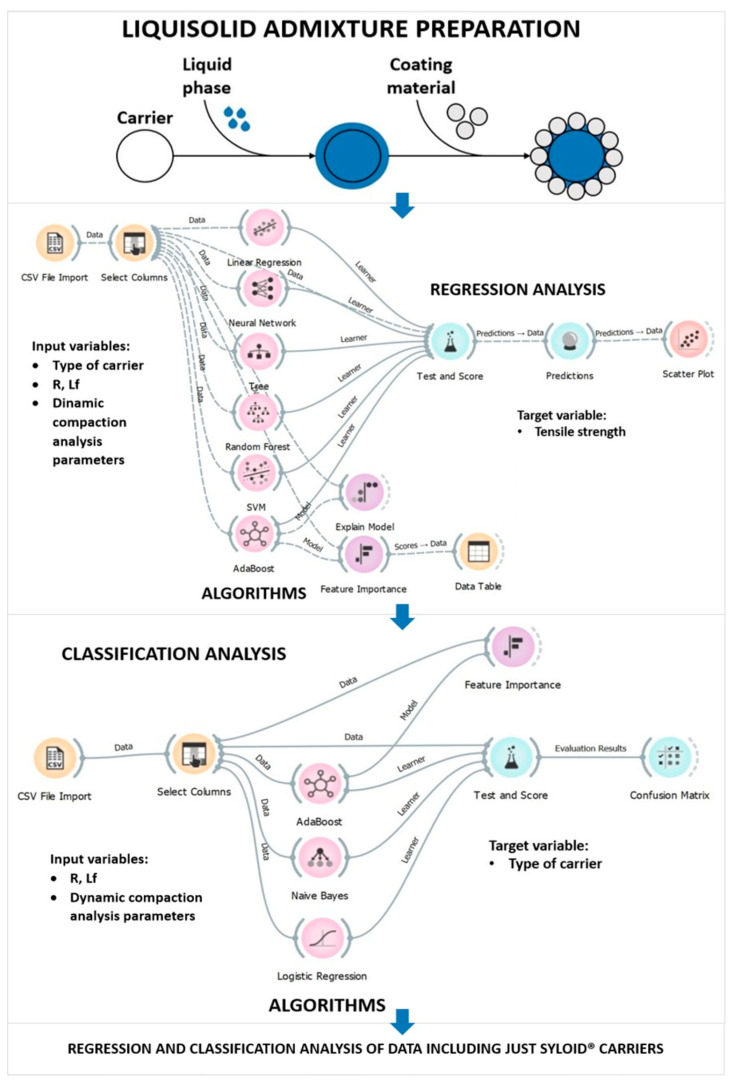
Flowchart of the proposed methodology.

In order to analyze the properties of LSS during compaction, a new data set was obtained by varying the compression load at 6 levels ranging from 250 to 500 kg with a 50 kg increment for each of the 25 compressed admixtures (marked with * in Table 1). The data set consisted of 9 variables with 150 entries each. The type of carrier used was defined as a categorical variable, while the remaining 8 variables were numerical and included the formulation factors R and Lf, as well as TS, CP, DS, ES, NWC, and ER. TS was chosen as the target variable, while the influence of the other variables on TS was investigated. A visual representation of the proposed methodology can be found in Figure 1.

First, a regression analysis was performed with the aim of determining whether TS could be predicted on the basis of some of the input variables. Six different algorithms were tested to select the one with the highest degree of prediction. The linear regression learning algorithm uses a linear function to model a relationship between independent and dependent variables, in this case input data and responses. It is one of the most commonly used regression learning algorithms because it is simple and relatively easy to train and interpret [51]. The multy-layer perceptron (MLP) algorithm is a type of feedforward neural network that consists of multiple layers of interconnected neurons that can develop non-linear functions between inputs and outputs. This type of neural network consists of at least three layers of neurons: input, hidden, and output layers, and each neuron has an activation function. The algorithm is trained using backpropagation, which means that the output data is sent back to check the prediction error, which improves the accuracy of the algorithm the more times the process is repeated [52]. Stochastic gradient descent (SGD) minimizes a loss function by approximating the gradient descent technique. Instead of considering the entire data set, only one sample at a time is considered, and based on the calculated gradient of the loss function, the model is updated. It has been used as an optimization function in MLP [53]. The decision tree algorithm works on the basis of splitting the data based on a common feature. The data set is represented by a root node, from which branching starts. It can be used for both regression and classification and for both numeric and categorical variables [54]. Random Forrest is an algorithm based on ensemble learning, which means that it improves the performance of the model by combining multiple classifiers, in this case, multiple decision trees. Each of the decision trees uses a sample of the data, and the final output is predicted by combining the results of each tree based on the majority of the predicted outputs [55]. AdaBoost is a boosting algorithm, meaning that it adjusts the weak learning units and improves their performance by combining their results into a weighted sum. It works for both classification and regression [56]. The Support Vector Machine (SVM) algorithm separates the data into classes by defining the hyperplane (decision boundary) based on the extreme cases/vectors, also called support vectors. This algorithm is used in both regression and classification analysis and can be used for both linearly and nonlinearly separated data [57]. Model validation was performed using the leave-one-out cross-validation method. A classification analysis was performed afterwards on the same set of data with the aim of determining the classification accuracy of the model in predicting the type of carrier used based on other numerical variables. Three algorithms were tested, including AdaBoost. Naive Bayes is a probabilistic classification algorithm, meaning it predicts how the data should be classified based on the probability that an object belongs to a particular class [58]. Logistic regression is a simple classification algorithm used to predict the outcome of a categorical dependent variable based on the independent variables. Both continuous and discrete data sets can be classified using this algorithm, but the result is always a categorical or discrete value (0 or 1) [59]. Lastly, a new data set that included only compressed LS formulations containing one of the two Syloid^®^ carriers used, Syloid^®^ XDP 3050 and Syloid^®^ XDP 3150, was analyzed in order to determine how differences in particle size affected the behavior of these LSS during compaction. The software settings for each of the algorithms can be found in Table S2 as part of the Supplementary Material.

## 3. Results and Discussion

### 3.1. Flowability of LS Admixtures

**Figure 2:**
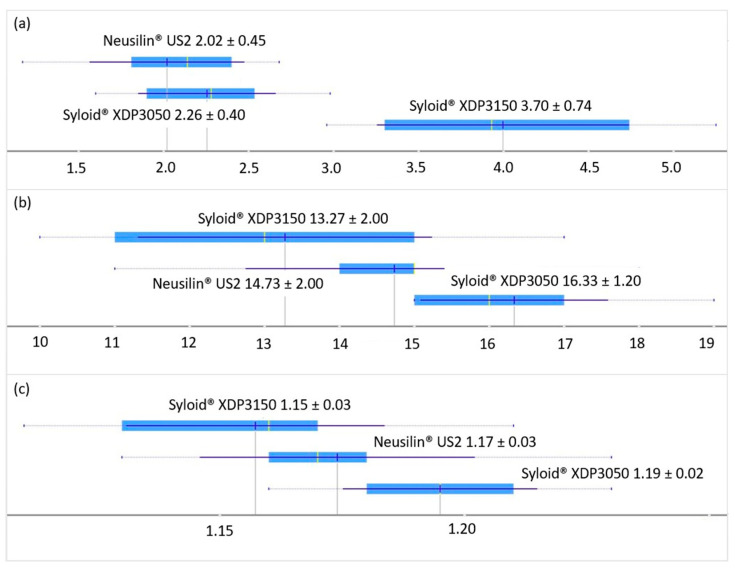
Flow properties of LS admixture presented as the mean value ± standard devation: (**a**) flow rate (g/s); (**b**) Carr index (%); (**c**) Hausner ratio.

Good flowability is one of the basic prerequisites for further optimization of LS formulations and can be expressed as flow rate, CI, or HR depending on the method used to determine it. The results of all three methods of determination are presented as the mean value and standard deviation in Figure 2. Regardless of the applied test method, a similar trend was noted, and the best flowability was observed in the admixtures prepared with the carrier Syloid^®^ XDP 3150. LS admixtures with the other two carriers showed slightly worse flowability, and the results were different when different testing methods were applied. Admixtures with Syloid^®^ XDP 3050 showed the highest values of CI and HR, while the lowest flow rate was recorded in admixtures containing Neusilin^®^ US2. However, t-test results showed that there was no statistically significant difference (*p* > 0.05) between the mean values of flow rates of admixtures with carriers Neusilin^®^ US2 and Syloid^®^ XDP 3050. The results confirmed that the difference in flowability between the two admixtures with Syloid^®^ carriers is statistically significant in the case of both testing methods (*p* < 0.001). Statistical significance (*p* < 0.001) was also observed between the mean values of the flow rate of admixtures prepared with Syloid^®^ XDP 3150 and Neusilin^®^ US2.

Based on the values of CI and HR and their corresponding descriptive terms proposed by the European Pharmacopoeia, the flowability of admixtures with Syloid^®^ XDP 3150 and Neusilin^®^ US2 carriers was described as good, and the flowability of admixtures with Syloid^®^ XDP 3050 was described as acceptable [47].

Pearson’s correlation showed that a low negative degree of correlation was present between formulation factors (R and L_f_) and flow rate, CI, and HR (correlation factor values less than −0.4) [60]. It was concluded that the categorical variable, the type of carrier used, had the most pronounced influence on the flow rate of the examined LSS; however, formulation factors (e.g., the amount of liquid phase that the system can incorporate) depend on the physico-chemical characteristics of the carrier itself (such as the size and shape of the particles, the SSA, and the diameter and volume of the pores).

In a study conducted by Kostelanska et al. [61], the structural and physical properties of multiple carriers were compared, including Neusilin^®^ US2 and Syloid^®^ XDP 3050. Neusilin^®^ US2 was found to be one of the best candidates for a carrier due to a very high SSA value (approximately 342 m^2^/g), which was higher than that stated by the manufacturer, and the pore volume of 0.69 cm^3^/g, which was among the highest of the carriers studied. SSA for Syloid^®^ XDP 3050 was determined to be approximately 289 m^2^/g while the pore volume was 0.58 cm^3^/g. Another study investigating mesoporous carriers determined that Syloid^®^ XDP 3150 had an SSA value of 295.7 m^2^/g [62]. The liquid load of all three carriers in the current study was in accordance with these results, Neusilin^®^ US2 was able to incorporate higher amounts of liquid phase (L_f_ values up to 1.3) compared to Syloid^®^ carriers (L_f_ values up to 1.0) while retaining good flowability. Several studies reported high values for the flowable liquid-loading potential of Neusilin^®^ US2 [63,64]. A recent study [26] showed that LS admixtures of the same composition (Neusilin^®^ US2, PEG 400, and Aerosil^®^ 200) with an L_f_ value of 0.55 and R values in the same range (10–20–30) had acceptable flowability with CI values from 14.63 to 17.71. In comparison to these results, the admixtures prepared in this research displayed good flowability with a CI mean value of 14.73 despite notably higher L_f_ values (0.7–1.3). These findings indicate that for porous carriers such as Neusilin^®^ US2, which are characterized by a very high SSA value, changes in formulation factors (specifically L_f_) do not have a notable effect on flowability and good flowability could be expected with the amounts of liquid phase that are even higher than those tested in the current study. Carrier particle size proved to be the attribute with the most notable influence on flowability. The particle size of the three carriers used was 150 µm, 50 µm, and 100 µm for Syloid^®^ XDP 3150, Syloid^®^ XDP 3050, and Neusilin^®^ US2, respectively [45,46]. The best flowability was observed in LS admixtures with the carrier Syloid^®^ XDP 3150, implying that admixtures with larger carrier particles flowed better, which was in accordance with the available literature data [65]. The results of the flow rate were also visualized by the decision tree. It was observed that the type of carrier was the parameter based on which the initial branching was made, while L_f_ was the next parameter with the greatest influence on the flowability of LS admixtures.

**Figure 3:**
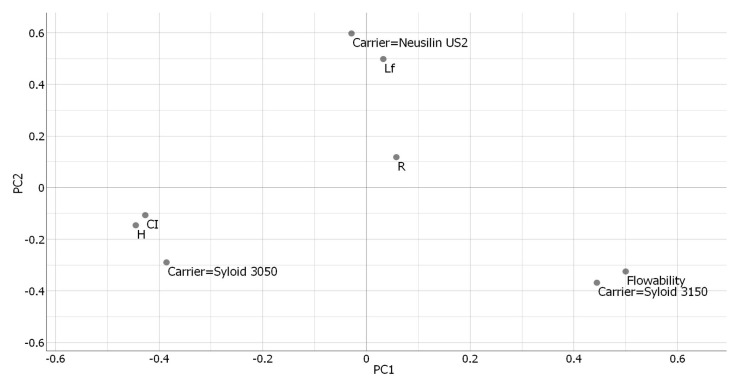
Results of principal component analysis for LS admixtures’ flowability results; PC1—first principal component; PC2—second principal component.

In order to better understand the mutual dependence of different parameters (type of carrier used, R, L_f_, flow rate, CI, and HR), multivariate data analysis was carried out using the principal component analysis (PCA). By applying this method, the number of data points is reduced by defining “principal components,” (PC) which, among other things, take into account the maximum variance originating from each of the variables [66]. The results of the PCA analysis are shown in Figure 3. It was noted that the first principal component (PC1) was able to explain approximately 41% of the variance and correlated to a higher degree with the 4 input variables (flow rate, CI, HR, and Syloid^®^ XDP 3150 carrier), with a positive correlation in the case of Syloid^®^ XDP 3150 carrier and flow rate and a negative one in the case of CI and HR, indicating that admixtures with carrier Syloid^®^ XDP 3150 achieved better flowability. The second principal component (PC2) was able to explain approximately 29% of the variance and was positively correlated with the carrier Neusilin^®^ US2 as well as with the L_f_, which was a result of LS formulations with this carrier having the highest amount of liquid phase incorporated within them.

**Figure 4:**
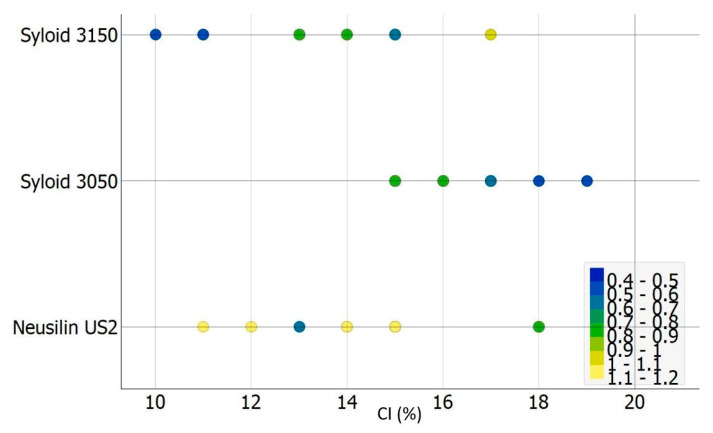
Carr index values depending on the carrier used. The color of the circles corresponds to liquid load factor (the colours follow the values given in the legend).

Figure 4 shows a scatter plot of the dependence of CI on the type of carrier used: L_f_ is shown in different colors according to the legend. It is interesting to notice that in the case of admixtures with Syloid^®^ XDP 3150 as the carrier, the increase in the amount of liquid phase in the system led to an increase in the CI value, i.e., a decrease in flowability. On the other hand, in the case of admixtures containing Syloid^®^ XDP 3050, there was a positive correlation between the increase in the amount of liquid phase and the indirect flowability. Although the flowability of the admixtures with Syloid^®^ XDP 3150 as a carrier was found to be better based on the mean values, this type of dependence should also be taken into account in further formulation considerations, as the aim with these systems is always to obtain admixtures with the highest possible proportion of liquid phase and consequently API, while maintaining good flowability.

### 3.2. Compaction Behavior of LSS

#### 3.2.1. Regression Analysis

A regression analysis was performed to establish a relationship between a set of independent variables and TS, which was selected as the dependent variable because it is one of the most significant indicators of the compacts’ mechanical characteristics. By establishing this connection, it would be possible to predict the outcomes, i.e., the TS of LS compacts could be predicted based on the tableting process parameters and/or the formulation factors. Several models were compared in order to find the one with the best ability to predict TS values based on the values of the remaining variables (Table 2).

Multiple models showed comparable performance with similar values of training and test errors. AdaBoost is the so-called “boosting algorithm,” meaning that it considers the weaknesses of other models and adjusts to improve performance accordingly [67]. Prediction with this model was highly accurate; however, there was a difference in accuracy depending on the carrier used. The highest accuracy was present for admixtures containing Syloid^®^ XDP 3050.

**Figure 5:**
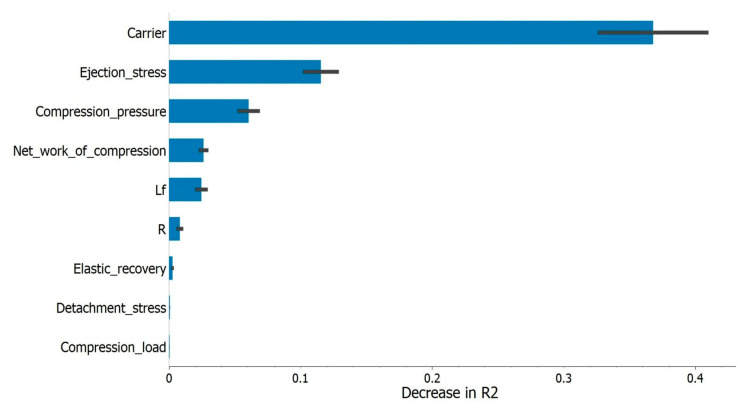
The influence of tested variables on regression model’s output (presented as the decrease in coefficient of determination that removing a variable would cause).

In addition to determining the best predictive model, one of the goals of the regression analysis was to determine which of the input variables were the most important for the model’s performance, i.e., to what extent removing one of the input variables affected the model’s predictive ability. The data obtained are shown in Figure 5.

**Figure 6:**
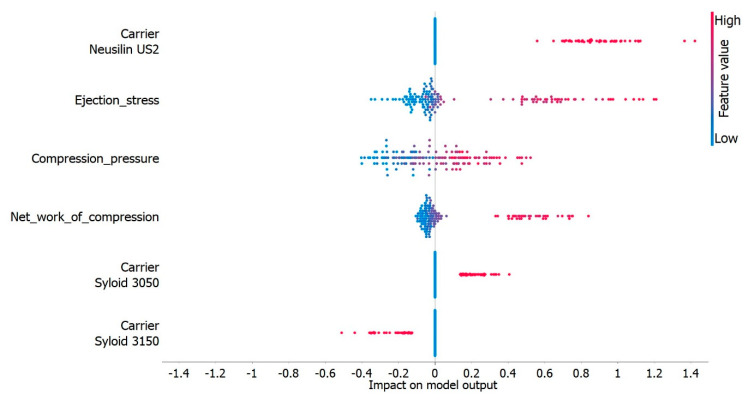
The impact of tested variables on regression model’s output.

The carrier type was the variable with the most notable impact on the model output, and further explanation of the model by the appropriate software features singled out carrier Neusilin^®^ US2 as the variable with the greatest impact (Figure 6). LS compacts containing this carrier achieved TS values that ranged from 1.65 to 4.34 MPa. According to the literature, data compacts with TS values higher than 1 MPa are acceptable [68]. LS compacts with the other two carriers achieved noticeably lower values of TS, 0.40 to 1.10 MPa and 0.50 to 2.00 MPa, for admixtures with Syloid^®^ XDP 3150 and Syloid^®^ XDP 3050, respectively. These results are in line with what has been reported in the literature. In a study investigating different mesoporous carriers, Mura et al. [62] compared the tablet crushing force of compacts prepared under the same conditions. With a value of 332.6 N, Neusilin^®^ US2 displayed superior compaction properties compared to Syloid^®^ XDP 3150 (tablet crushing force of 13.4 N). Due to its high absorption capacity, Neusilin^®^ US2 has been widely used as a carrier in lipid-based drug delivery systems as well. Gumaste et al. [69] reported that Neusilin^®^ US2 was able to absorb the lipid component of the formulation at a ratio of 1:1 and still retain a TS higher than 1 MPa despite the type of liquid used or CP applied (45–270 MPa). Despite the highest liquid load, the TS of the LS compacts with Neusilin^®^ US2 was the highest of the three carriers tested. These findings highlighted the importance of selecting a carrier with suitable characteristics, such as a high SSA, and indicated that the properties of the carrier itself were largely responsible for the behavior of LSS during compaction, while other formulation factors contributed less. These results also highlight Neusilin^®^ US2 as the carrier that exhibits both good flowability and good compressibility while retaining large amounts of the liquid phase.

Among the parameters specific to the tableting process, ES and CP stood out as the variables with the most prominent impact on the model’s performance (Figure 5). Both of these parameters can be monitored throughout the tableting process, which meant that by establishing a suitable model with TS as a dependent variable, it would be possible to predict the TS of LS compacts during the production process, which is important from the perspective of the pharmaceutical industry, which relies on approaches such as quality by design (QbD) and the application of different process analytical technology (PAT) tools in order to obtain a product of consistent quality. Application of the QbD approach in the case of LS technology would consider understanding critical material attributes (e.g., carrier particle size, flowability, bulked and tapped density) and critical process parameters (e.g., mixing time and speed, CP, compression speed), as well as their impact on critical quality attributes such as drug content, disintegration time, dissolution, tablet hardness, etc. The PAT approach implies constant monitoring of different parameters in real time, which, together with the implementation of control strategies, enables obtaining products of the required quality while saving time and resources, and as a result, gives an opportunity for the use of a real time release strategy [70].

The ES values (Table S2) in the case of both Syloid^®^ carriers were within acceptable limits (lower than 5 MPa) [71]. On the other hand, the compacts with Neusilin^®^ US2 showed higher values of this parameter regardless of the applied compression pressure, indicating poor lubricative properties of the material and potential problems that could occur during tableting due to the adhesion of the material to the tableting equipment. However, it was observed that with the increase in the amount of the liquid phase, the value of ES decreased. The improved lubrication effect could be caused by the small amounts of liquid phase that were squeezed out by compression and settled on the surface of the particles.

Depending on the type of carrier used, changes in CP affected TS to a greater or lesser extent (Figure 6), which could be explained by the differences in the behavior of different materials during compression. This was confirmed by examining the dependence of TS on CP, where a positive correlation was observed between these two parameters for both Syloid^®^ carriers, which meant that with an increase in CP, the TS of the compacts also increased. In the case of the LS admixtures with Neusilin^®^ US2, changes in CP did not significantly affect TS. This indicated that this carrier was more robust during tableting, which is a positive characteristic of the material and allows more flexibility in the development of the production process. Even though the TS of the LS compacts with Neusilin^®^ US2 decreased with increasing liquid load, the values obtained, which were around 2 MPa, were still acceptable and higher than those obtained in LS compacts with the other two carriers in their composition at the same or even higher CPs.

#### 3.2.2. Classification Analysis

The classification analysis, as one of the basic tools in ML, is based on the idea of dividing data into multiple groups based on some common characteristic. Type of the carrier used in the formulation proved to be one of the most significant parameters affecting flowability and compaction of LSS. Therefore, the goal was to utilize continuous data (L_f_, R, TS, CP, DS, ES, NWC, and ER) for carrier-based classification. Three models were tested, and the prediction accuracy of the models was assessed based on the parameters shown in Table 3. The AdaBoost algorithm, which can be used for both regression and classification analysis, proved to be the best choice in this case as well.

**Figure 7:**
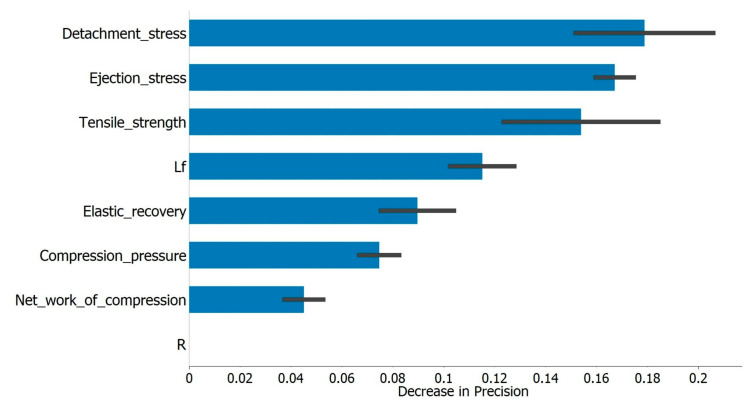
The influence of tested variables on classification model’s output (presented as the decrease in precision that removing a variable would cause).

The degree of prediction of the AdaBoost algorithm was illustrated by the so-called confusion matrix, a matrix showing the percentage of correctly predicted cases. The developed model had the highest number of correct predictions for admixtures with Neusilin^®^ US2 (98.1%) and a higher percentage of prediction error for admixtures containing Syloid^®^ carriers (the percentage of correct predictions was 86.9% for Syloid^®^ XDP 3050 and 83.3% for Syloid^®^ XDP 3150). Figure 7 represents the influence of the tested parameters on the model through the decrease in precision that removing certain parameters would cause. DS, ES, and TS had the greatest influence on the model precision, which is in accordance with some of the conclusions previously established through regression analysis. Both TS and ES values were higher in the case of Neusilin^®^ US2 as the carrier, resulting in less overlap and more precise classification by the model. In addition, in this phase of the research, excipients such as lubricants were not added to the LS admixtures; therefore, the parameters DS and ES were influenced primarily by the characteristics of the carrier itself, which would also explain why these were the parameters with the most significant influence on the model prediction.

#### 3.2.3. Analysis of Differences between Syloid^®^ Carriers

Previously conducted classification analysis showed lower prediction accuracy of the model for LSS with Syloid^®^ carriers, which could be a consequence of their similarities and the fact that these two carriers are chemically the same, with the main difference between them being the particle size.

**Figure 8:**
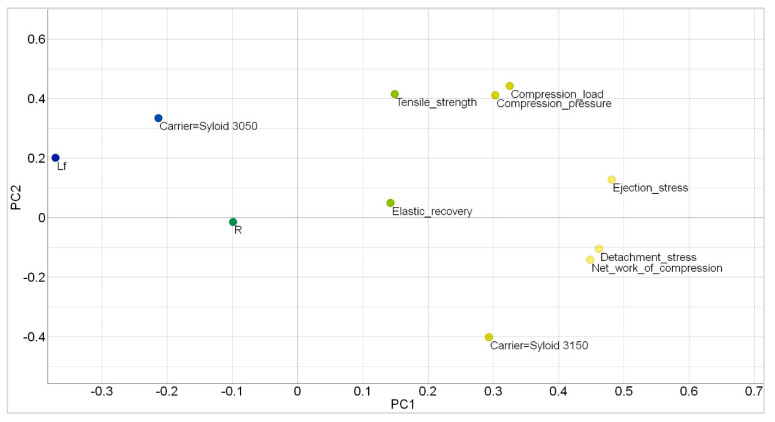
Results of principal component analysis for two Syloid^®^ carriers.

The differences between the two carriers could not be observed graphically by displaying the dependence of TS on one of the tableting process parameters. This led to data analysis using PCA, which allowed a clear division between the two carriers and managed to explain approximately 65% of the variance (~39% was explained by PC1 and ~26% was explained by PC2). The positive correlation between carrier Syloid^®^ XDP 3050 and L_f_, which were both negatively correlated with PC1 was observed (Figure 7). On the other hand, higher values of PC1 indicated a sample that achieved higher values of DS, ES, and NWC and contained Syloid^®^ XDP 3150 as a carrier. Following PC2, a positive correlation between Syloid^®^ XDP 3050 and TS was also observed (Figure 8). From these results, it was concluded that Syloid^®^ XDP 3150 had slightly worse behavior during compression and that compacts with this carrier achieved higher values of DS, ES, and NWC and lower values of TS.

**Figure 9:**
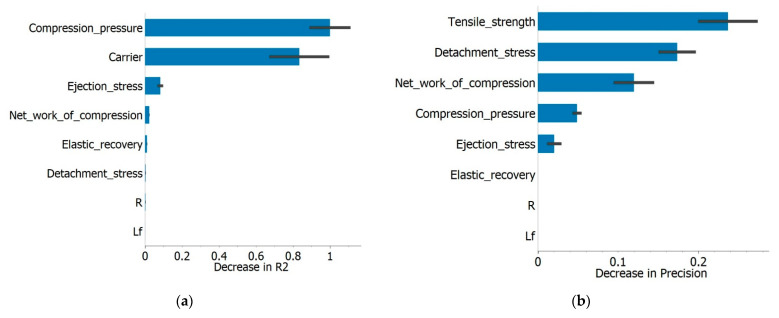
Analysis of differences in compaction properties between Syloid^®^ carriers: (**a**) The influence of tested variables on regression model’s output with tensile strength as dependant variable (presented as the decrease in coefficient of determination that removing a variable would cause).; (**b**) The influence of tested variables on classification model’s output (presented as the decrease in precision that removing a variable would cause).

The additional regression and classification analysis performed on the data obtained for formulations with two Syloid^®^ carriers confirmed some of the previously derived conclusions. In both cases, AdaBoost was found to be the algorithm with the highest accuracy of model output (R^2^ in regression analysis: 0.891, precision in classification analysis: 0.833). CP and the type of carrier were singled out as the most significant parameters for model prediction in the regression analysis (Figure 9a). These results are consistent with the previous conclusion that there is a high degree of correlation between TS and CP in LSS with Syloid^®^ carriers. It is interesting that ES was not a parameter that had a pronounced influence on the model in this case, which led to the conclusion that this was only the case for admixtures with Neusilin^®^ US2. TS, CP, and NWC were the parameters that had the greatest influence on the classification model’s predictive ability, which was in accordance with the conclusions from the PCA and regression analysis results (Figure 9b).

## 4. Conclusions

Maintaining the balance between increasing the amount of liquid phase and consequently the amount of API in the LS system while maintaining good flowability and compaction properties is one of the most common problems faced during the formulation of the LSS. The main advantage of using ML tools in the development of LS formulations is that their application allows a more detailed and comprehensive insight into potential problems and enables easier observation and description of the complex relationships between individual variables.

Additionally, it was concluded that the type of carrier had the strongest influence on the flowability of LS admixtures. The admixtures with the carrier Syloid^®^ XDP3150 showed the best flowability, while the flowability of the admixtures with the other two carriers was slightly lower but still within acceptable limits. The type of carrier proved to be one of the parameters with the greatest influence on the TS of LS compacts. Neusilin^®^ US2 stood out as a good choice because, even with an L_f_ value of 1, the values of TS were satisfactory and were not affected by changes in CP. Compacts containing Syloid^®^ XDP3050 as the carrier showed slightly higher TS values than compacts with Syloid^®^ XDP3150 as the carrier. Monitoring the values of tableting process parameters proved promising for predicting TS. In the case of the Neusilin^®^ US2, ES was shown to be the variable that significantly affected the model’s prediction, while in the case of the Syloid^®^ carrier, CP had the greatest influence. The AdaBoost algorithm proved to be the best choice for building predictive models for both regression and classification analysis with high predictive accuracy—the coefficient of determination for regression analysis was 0.94, while the precision of the model for classification analysis was 0.90. The analysis of the differences between the two Syloid^®^ carriers showed the importance of particle size.

Furthermore, the application of the ML tools proved to be a useful approach in the development of LSS, providing a more comprehensive insight into the flowability and compaction properties while also contributing to a better understanding of the whole tableting process and all its phases, which is the basis of the QbD approach. The developed models showed a high degree of success in predicting the given outcomes and could form the basis for further research with selected formulations. The results presented in this paper provide insight into how ML can be incorporated into the development and optimization of LSS, which will hopefully help the application of ML reach its full potential in this research area.

## Figures and Tables

**Table 1:** Composition of prepared LS admixtures and their flow properties.

Carrier	R ^1^	L_f_ ^2^	PEG (%)	Flow Rate (g/s)	CI ^3^ (%)	HR ^4^
Neusilin^®^ US2	10 *	0.7	38.9	1.81 ± 0.09	14.6 ± 0.6	1.17 ± 0.01
	10 *	0.8	42.1	1.81 ± 0.03	15.0 ± 1.4	1.18 ± 0.02
	10 *	1.0	47.6	2.40 ± 0.03	13.9 ± 1.7	1.16 ± 0.02
	10	1.2	52.2	2.30 ± 0.06	14.2 ± 1.4	1.17 ± 0.02
	10	1.3	54.2	2.05 ± 0.11	13.9 ± 1.0	1.16 ± 0.01
	20 *	0.7	40.0	1.23 ± 0.03	18.3 ± 0.2	1.22 ± 0.00
	20 *	0.8	43.2	1.30 ± 0.03	18.4 ± 0.8	1.23 ± 0.01
	20 *	1.0	48.8	2.22 ± 0.06	15.2 ± 1.8	1.18 ± 0.03
	20	1.2	53.3	2.68 ± 0.04	11.4 ± 0.1	1.13 ± 0.00
	20	1.3	55.3	2.43 ± 0.05	14.0 ± 0.9	1.16 ± 0.01
	30 *	0.7	40.4	2.14 ± 0.11	12.7 ± 1.4	1.15 ± 0.02
	30 *	0.8	43.6	1.17 ± 0.02	18.2 ± 0.8	1.22 ± 0.01
	30 *	1.0	49.2	2.02 ± 0.08	14.8 ± 1.8	1.17 ± 0.03
	30	1.2	53.7	2.42 ± 0.14	12.2 ± 1.1	1.14 ± 0.01
	30	1.3	55.7	2.32 ± 0.04	14.5 ± 2.8	1.17 ± 0.04
Syloid^®^ XDP 3150	10 *	0.6	35.3	4.48 ± 0.49	15.0 ± 1.0	1.17 ± 0.01
	10 *	0.7	38.9	3.46 ± 0.20	15.2 ± 1.8	1.18 ± 0.02
	10	0.8	42.1	3.30 ± 0.26	14.2 ± 1.0	1.17 ± 0.01
	10	1.0	47.6	4.75 ± 0.37	17.0 ± 0.0	1.21 ± 0.00
	20 *	0.6	35.3	4.50 ± 0.68	10.0 ± 0.0	1.11 ± 0.00
	20 *	0.7	38.9	3.31 ± 0.19	14.0 ± 2.0	1.17 ± 0.03
	20	0.8	43.2	3.28 ± 0.39	13.2 ± 0.2	1.15 ± 0.00
	20	0.9	46.2	4.74 ± 0.49	11.0 ± 0.0	1.13 ± 0.00
	30 *	0.6	36.7	5.25 ± 0.50	11.0 ± 1.0	1.13 ± 0.01
	30 *	0.7	40.4	3.93 ± 0.54	13.0 ± 1.0	1.16 ± 0.02
	30	0.8	43.6	2.96 ± 0.26	12.7 ± 1.1	1.15 ± 0.01
Syloid^®^ XDP 3050	10	0.5	31.3	2.43 ± 0.32	19.0 ± 1.0	1.23 ± 0.02
	10 *	0.6	35.3	2.77 ± 0.23	18.0 ± 0.0	1.22 ± 0.01
	10 *	0.7	38.9	2.40 ± 0.22	17.0 ± 0.0	1.21 ± 0.00
	10 *	0.8	42.1	1.60 ± 0.04	16.2 ± 0.7	1.19 ± 0.01
	10	1.0	47.6	2.98 ± 0.19	15.0 ± 1.0	1.18 ± 0.01
	20 *	0.6	35.3	1.95 ± 0.24	17.0 ± 0.0	1.20 ± 0.00
	20 *	0.7	38.9	1.80 ± 0.01	16.0 ± 2.0	1.20 ± 0.04
	20 *	0.8	43.2	1.85 ± 0.02	15.7 ± 1.0	1.19 ± 0.01
	20 *	0.9	46.2	2.53 ± 0.10	15.0 ± 1.0	1.17 ± 0.01
	30 *	0.6	36.7	2.54 ± 0.36	15.0 ± 2.0	1.16 ± 0.01
	30 *	0.7	40.4	2.16 ± 0.04	17.0 ± 1.0	1.21 ± 0.02
	30 *	0.8	43.6	2.05 ± 0.09	15.0 ± 2.0	1.18 ± 0.03

^1^ carrier to coating material ratio; ^2^ liquid load factor; ^3^ Carr index; ^4^ Hausner ratio; * LS admixtures that were compressed in order to analyze the properties of LSS during compaction.

**Table 2:** Models tested during regression analysis.

Model	MSE ^1^		RMSE ^2^		MAE ^3^		R^2 4^	
	Training Data	Test Data	Training Data	Test Data	Training Data	Test Data	Training Data	Test Data
AdaBoost	0.00015201	0.06507389	0.01232917	0.25509584	0.00312455	0.17468138	0.99986102	0.94050451
SVM ^5^	0.03555609	0.06479570	0.18856323	0.25454999	0.12102410	0.16794962	0.96749192	0.94075885
Linear Regression	0.06839292	0.08120252	0.26152040	0.28496056	0.19852719	0.21471604	0.93746999	0.92575848
Neural networks	0.05815805	0.07246392	0.24115981	0.26919124	0.18134422	0.20151931	0.94682749	0.93374798
Tree	0.00749373	0.08475951	0.08656636	0.29113486	0.06079310	0.19475213	0.99314866	0.92250642
Random forest	0.06050200	0.08642901	0.24597155	0.29398810	0.18556466	0.23168898	0.94468447	0.92098003

^1^ mean square error; ^2^ root mean square error; ^3^ mean absolute error; ^4^ coefficient of determination; ^5^ support vector machine.

**Table 3:** Models tested during classification analysis.

Model	AUC ^1^		CA ^2^		Precision		Recall	
	Training Data	Test Data	Training Data	Test Data	Training Data	Test Data	Training Data	Test Data
AdaBoost	1.00000000	0.92381536	1.00000000	0.90000000	1.00000000	0.90213558	1.00000000	0.92381536
Naïve Bayes	0.97528595	0.9620098	0.87333333	0.86666667	0.88611765	0.87748252	0.87333333	0.9620098
Logistic Regression	0.92279412	0.90767974	0.78666667	0.78666667	0.78529412	0.78529412	0.78666667	0.90767974

^1^ area under curve, ^2^ classification accuracy.

## Data Availability

The data presented in this study are available on request from the corresponding author. Interested readers may access the paper-related repository on GitHub via the following link: https://github.com/jelenadjuris/liquisolid_ML_paper (accessed on 10 January 2023).

## Supplementary Materials

The following supporting information can be downloaded at: https://www.mdpi.com/article/10.3390/pharmaceutics15030741/s1. Table S1. Modeling aspects for each of the tested models. Table S2: Composition of liquisolid admixtures and values of tableting process parameters.
