# Supplementary material for: Application of Machine-Learning Algorithms for Better Understanding the Properties of Liquisolid Systems Prepared with Three Mesoporous Silica Based Carriers
Source: Pharmaceutics. 2023 Feb 23;15(3):741. doi: 10.3390/pharmaceutics15030741 (PMC10054079; doi:10.3390/pharmaceutics15030741)
Supplement: Supplementary file 1 [file pharmaceutics-15-00741-s001.zip › pharmaceutics-2193251-supplementary.pdf]

Table S1. Modeling aspects for each of the tested models.

Model	Modeling aspects
Linear Regression	Regularization - L1 Lasso regression, α=0.0001; default data preprocessor.
Neural networks	100 neurons in the hidden layers, activation function - Rectified Linear Unit (ReLU), solver – Stohastic Gradient Descent, Regularization - α=0.0001, maximal number of iterations – 200; default data preprocessor.
Decision Tree	Min. number of instances in leaves – 2, do not split subsets smaller than 5, maximal three depth 100; stop classification when majority reaches 95%.
Random forest	Number of trees – 10, limit depth of individual trees – 3, do not split subsets smaller than 5; default data preprocessor.
Support Vector Machine	Cost (C) – 1, regression loss epsilon (ε) – 0.10; Kernel – RBF; numerical tolerance – 0.001, iteration limit – 100; default data preprocessor.
AdaBoost	Base estimator – tree, number of estimators – 50, learning rate – 1.0; Boosting method: Regression loss function – exponential, Classification algorithm – SAMME.R; default data preprocessor.
Naïve Bayes	Default data preprocessor.
Logistic Regression	Regularization - L1 Lasso regression, C=0.600; default data preprocessor.

Table S2. Composition of liquisolid admixtures and values of tableting process parameters.

Carrier	R [1]	L_f [2]	PEG (%)	CP[3] (MPa)	TS[4] (MPa)	DS[5] (MPa)	ES[6] (MPa)	NWC[7] (J)	ER[8] (%)
Neusilin® US2	10	0.7	38.9	186.9 ± 0.3 [9]	2.5 ± 0.6	1.6 ± 0.1	10.2 ± 1.2	2.8 ± 0.1	46.3 ± 1.3
	10	0.8	42.1	189.2 ± 0.5	2.9 ± 0.6	2.3 ± 0.2	9.0 ± 0.6	2.4 ± 0.0	42.2 ± 1.9
	10	1.0	47.6	190.1 ± 2.6	1.7 ± 0.5	1.2 ± 0.2	7.8 ± 0.8	1.5 ± 0.0	36.9 ± 3.7
	20	0.7	40.0	190.3 ± 1.3	3.8 ± 0.5	1.6 ± 0.0	11.7 ± 1.1	2.5 ± 0.1	45.7 ± 3.9
	20	0.8	43.2	191.0 ± 0.8	3.1 ± 0.4	2.6 ± 0.0	9.3 ± 0.4	2.2 ± 0.0	41.5 ± 1.9
	20	1.0	48.8	188.2 ± 1.6	2.0 ± 0.1	1.8 ± 0.3	7.3 ± 0.4	1.4 ± 0.1	35.4 ± 3.5
	30	0.7	40.4	190.3 ± 0.3	4.3 ± 0.3	1.9 ± 0.2	11.3 ± 0.5	2.5 ± 0.0	42.4 ± 0.5
	30	0.8	43.6	191.3 ± 0.4	2.8 ± 0.4	2.5 ± 0.1	8.8 ± 0.1	2.3 ± 0.0	42.7 ± 1.8
	30	1.0	49.2	192.5 ± 0.1	2.2 ± 0.4	2.3 ± 0.1	8.4 ± 0.6	1.4 ± 0.0	43.8 ± 0.9
Syloid® XDP 3150	10	0.6	35.3	175.7 ± 0.4	1.1 ± 0.2	4.8 ± 0.3	5.1 ± 0.1	1.6 ± 0.1	43.7 ± 1.9
	10	0.7	38.9	177.9 ± 0.2	1.1 ± 0.1	4.7 ± 0.2	5.3 ± 0.3	1.3 ± 0.0	36.4 ± 1.0
	20	0.6	35.3	176.2 ± 0.2	0.9 ± 0.1	6.5 ± 0.2	6.9 ± 0.2	1.5 ± 0.0	39.6 ± 0.9
	20	0.7	38.9	178.1 ± 0.3	0.9 ± 0.1	4.5 ± 0.3	5.6 ± 0.2	1.2 ± 0.0	36.2 ± 0.7
	30	0.6	36.7	176.5 ± 0.1	1.0 ± 0.1	4.1 ± 0.1	4.7 ± 0.1	1.5 ± 0.0	42.4 ± 1.8
	30	0.7	40.4	178.3 ± 0.3	1.0 ± 0.0	3.1 ± 0.0	4.3 ± 0.1	1.2 ± 0.0	49.4 ± 2.3
Syloid® XDP 3050	10	0.6	35.3	176.1 ± 0.3	1.9 ± 0.3	4.5 ± 0.3	5.2 ± 0.1	1.5 ± 0.1	41.4 ± 1.0
	10	0.7	38.9	178.2 ± 0.1	1.8 ± 0.1	3.1 ± 0.2	4.8 ± 0.1	1.2 ± 0.0	34.7 ± 0.8
	10	0.8	42.1	181.3 ± 0.3	1.5 ± 0.1	2.6 ± 0.0	3.7 ± 0.1	0.9 ± 0.0	53.3 ± 2.3
	20	0.6	35.3	176.6 ± 0.1	2.0 ± 0.1	4.5 ± 0.3	5.2 ± 0.1	1.4 ± 0.0	50.3 ± 1.1
	20	0.7	38.9	178.9 ± 0.2	1.8 ± 0.1	4.7 ± 0.1	6.3 ± 0.1	1.2 ± 0.0	35.3 ± 1.2
	20	0.8	43.2	183.4 ± 0.1	1.4 ± 0.0	2.4 ± 0.0	3.3 ± 0.1	0.8 ± 0.0	51.5 ± 5.0
	20	0.9	46.2	189.9 ± 0.6	1.0 ± 0.0	1.5 ± 0.0	2.8 ± 0.1	0.4 ± 0.0	27.1 ± 1.9
	30	0.6	36.7	176.2 ± 0.0	1.9 ± 0.2	4.3 ± 0.3	5.1 ± 0.0	1.5 ± 0.0	41.3 ± 0.2
	30	0.7	40.4	180.0 ± 0.2	1.8 ± 0.3	2.8 ± 0.0	4.3 ± 0.1	1.1 ± 0.0	31.7 ± 1.7
	30	0.8	43.6	184.2 ± 0.2	1.3 ± 0.1	2.4 ± 0.0	3.2 ± 0.1	0.7 ± 0.0	53.4 ± 0.4

[1] carrier to coating material ratio; [2] liquid load factor; [3]compression pressure; [4]tensile strength; [5]detachment stress; [6]ejection stress; [7]net work of compression; [8]elastic recovery; [9]all the values are presented as mean value ± standard deviation obtained during compaction at a compression load of 500kg.
